# Case of Umbilical Hernia, with Loss of Portion of Intestine

**Published:** 1874-06

**Authors:** J. N. Reeder

**Affiliations:** Lacon, Ill.


ARTICLE II.—*A Case of Strangulated Umbilical Hernia, with Loss of Portion of Intestine.* By J. N. REEDER, M.D., Lacon, Ill.

On the 14th of February, 1871, I was called to see Mrs. Norman Fenn, of this city, aged about 68 years. I found her bathed in a cold, clammy perspiration, her pulse very feeble and frequent, her stomach excessively irritable, constantly retching, though her ejections were little else than the fluids she drank, and were of a distinctly stercoraceous odor; in short, her general appearance, expression of countenance, etc., were indicative of fast approaching collapse. She complained of no acute pain, but an indescribable distress—a sense of sinking, as she described it. Her stomach not being in a condition to retain medicine, I prepared and gave, hypodermically, three grs. sulph. quinine and one-fourth gr. sulph. morphine. My object in giving quinine in this case was for its antiseptic property, feeling that her great depression was owing in a large degree to a septic condition of the blood. In fifteen minutes the injection was repeated, with the effect of bringing up the pulse, causing general warmth of the surface, relieving her distress—in short, of establishing very perfect reaction.

Upon examination I found an immense "umbilical hernia," as large as a child's head, and as tympanitic as a blown bladder, the integuments covering the tumor being highly inflamed, of a dark livid color, and on the verge of giving way from excessive distention. In answer to my inquiry, why assistance had not been called sooner, I was told that she had been for thirty years incumbered with the hernia; that she had had frequent attacks similar to this; that she had always before been able to relieve herself by fomentations, and such means as she had at hand, and that she did not realize the danger she was in at such times. I learned farther that the strangulation had occurred three days since, and that nothing had been done for her relief except what the family had been able to do, though she had suffered the agonies of death. Of course my only alternative was an artificial anus for present relief. Being fully satisfied that there existed adhesions between the abdominal wall and the protruding loop of intestine, sufficiently strong to prevent the escape of fæcal matter, or the receding of the gangrenous intestine into the peritoneal cavity (the hernia having been irreducible for many years), I made an opening through the integuments and tissues beneath, exposing the intestine, which gave way from distention as soon as the support of the integuments was removed, giving exit to the gaseous and feculent contents of the loop. I then enlarged the incision in the integuments, exposing fully the interior of the tumor, which I found, as I expected, in a gangrenous condition, a well defined line of demarcation appearing at the point of exit, or opening in the abdomen. I proceeded carefully to separate the gangrenous mass at this line, and removed the whole contents of the sack, introducing into the two openings at the bottom, tents of soft sponge prepared in carbolized cocoa butter, applying a compress and bandage around the body. Every distressing symptom disappeared immediately on the escape of the pent-up contents of the included loop, and, under the use of quinine and morphine, she rapidly improved, and the integumentary covering of the hernia sloughed away, leaving an ugly looking ulcer of five inches in diameter, which healed down to the openings in the abdomen by granulation. Upon examining the mass removed I found that I had taken away fourteen inches of the transverse colon, with a large portion of omentum. During the healing the two openings were kept corked with sponge tents prepared as above, the upper one to prevent the constant escape of fæces, and the lower for the purpose of keeping it freely open, with the view of facilitating the operation I had in contemplation for the closure of the disgusting artificial anus, which I successfully accomplished some two months after the removal of the loop.

The first step in the operation was to remove the spur-like process between the upper and lower openings, which I attempted to do by passing a needle and ligature from one orifice to the other, but owing to the thickness of the abdominal walls and the fear that I might include within the stitch some portion of small intestine or other vital structure, I abandoned the plan, and adopted the long jawed forceps. Having no suitable instrument, I set myself to work to get one up for the occasion. Cutting the finger loops from a strong polypus forceps, having the arms brought together for jaws, and having a small rod with thumb screw upon it passed through holes drilled through the points of the short jaws, I had an instrument which answered my purpose perfectly. Passing one arm of my clamp into each of the open mouths their full length (four inches), I turned the screw till some pain was complained of.

Every day a few turns of the screw were added, till the tenth day it was found to have cut its way through, and was removed. Very little pain or tenderness was complained of from first to last. I had now a perfect cut-off, and was ready for the closure, which I attempted to accomplish by inserting, after having pared the margins of the orifice, common silk sutures—but failed, as I expected. My second attempt was successful. After thoroughly cleansing the bowel by injections of warm water, I carefully freshened the opening again, from the integumentary margin down to the peritoneal cavity. Arming a large curved needle with silver wire, I inserted it through the integuments fully an inch from the margin of the opening. Introducing one finger of my left hand as a guide to the point of the needle, I passed it down to the inner edge, and out on the opposite side at a corresponding distance from the margin. Three of these wires were passed through in the same manner, and were brought through small porcelain buttons for the purpose of relieving the strain of the wire upon the integuments, and then drawn sufficiently tight by twisting, to bring the bottom of the orifice into perfect apposition; the outer edge was drawn together by common adhesive plasters, warm water compresses and a bandage around the body, as in the first attempt; the patient was kept on her back and opium given freely to secure rest and keep the bowels quiet. No solid food was allowed. Considerable inflammation followed this procedure, but by adding tr. arnica to the fomentations, it soon subsided. After about a week the wires were removed, and union found to have taken place except a small point not larger than a broom straw, through which a little gas would occasionally escape, but nothing more. This little fistula closed of its own accord in the course of a few weeks. For ten days after the closure no discharge was had through the bowels, though no inconvenience was felt in consequence. Injections of warm water were given per anum for the purpose of dilating the long idle rectum and descending colon, and to stimulate the bowels to natural action, which was soon effected. Their action, however, was feeble for some weeks, but by the occasional aid of injections they began to act freely and naturally. It is now over two years since the closure. She has suffered no pain nor inconvenience in that region, is in perfect health, does her own housework, and in fact is in better health than she had been for many years before the accident.

I have been careful to give the case in detail as it occurred, and was treated, not stopping to discuss the propriety or impropriety of the steps taken or the means used in its management, leaving others to do that.

